# *Actinomyces meyeri* Popliteal Cyst Infection and Review of the Literature

**DOI:** 10.1155/2017/9704790

**Published:** 2017-01-31

**Authors:** Bharath Raj Palraj, Ala S. Dababneh

**Affiliations:** Division of Infectious Diseases, Mayo Clinic 200, First Street SW, Rochester, MN 55905, USA

## Abstract

A 66-year-old, Caucasian male presented with pain and swelling involving the left knee of one-week duration. Arthrocentesis was negative for evidence of septic arthritis. Magnetic resonance imaging (MRI) study of the left knee showed degenerative arthritis, partial tear of medial meniscus, and a complex fluid collection along the posteromedial aspect of the left knee suggestive of popliteal cyst. He underwent arthroscopy with partial medial meniscectomy. Intraoperative joint fluid was noted to be cloudy but cultures were negative. Arthroscopic procedure provided him with temporary relief but the pain and swelling in the posterior aspect of the left knee recurred in 6 weeks. Repeat MRI showed complex fluid collection in the posterolateral aspect of left knee. Ultrasound guided aspiration of the fluid collection revealed purulent material and cultures grew* Actinomyces meyeri*. He was treated with 6 weeks of intravenous penicillin regimen followed by 18 months of oral penicillin.

## 1. Background

*Actinomyces meyeri* is part of oral microflora and a very uncommon cause of infection in humans. Only 34 cases of infections due to* A. meyeri* have been reported in English language literature, when search was done in December 2016. The majority of cases were pneumonia, empyema, osteomyelitis, and abscesses in liver, spleen, and brain. Popliteal cyst (Baker's cyst) infection is a very rare complication that is usually associated with septic arthritis. To our knowledge, this is the first report of a popliteal cyst infection due to* Actinomyces meyeri* in the English language medical literature. Based on clinical case reports, it requires prolonged antibiotic treatment along with surgical drainage procedure to cure this infection.

## 2. Case Presentation

A 66-year-old Caucasian male with a past medical history of mild degenerative joint disease presented to his primary physician with pain and swelling of the left knee without any history of recent injury. He did not have any history of alcohol abuse but noted history of dental abscess that was treated with root canal procedure approximately 4 months prior to the initial presentation. On physical examination, he had left knee joint effusion and about 35 ml of clear joint fluid was aspirated. Unfortunately, joint fluid was not sent for analysis or culture. Patient was diagnosed with possible degenerative arthritis with effusion and intra-articular steroid injection was given to reduce inflammation and alleviate pain. After a few days, patient presented with recurrence of left knee pain and progressive swelling in the left calf along with intermittent chills. On examination, he had left knee effusion, probable popliteal cyst along with erythema, tenderness, and induration in the calf region. Ultrasound noted a 6 × 2 × 3 cm complex fluid collection along the posteromedial aspect of left knee, suggestive of popliteal cyst with debris or hemorrhage. Magnetic resonance imaging (MRI) of his left knee confirmed the ultrasound findings and showed large radial tear through medial meniscus body, anterior cruciate ligament tear. Patient underwent left knee arthroscopy with partial medial meniscectomy, chondroplasty, and subtotal synovectomy. Intraoperatively, the joint fluid was noted to be cloudy but both aerobic and anaerobic cultures of joint fluid were negative.

**Figure 1:**
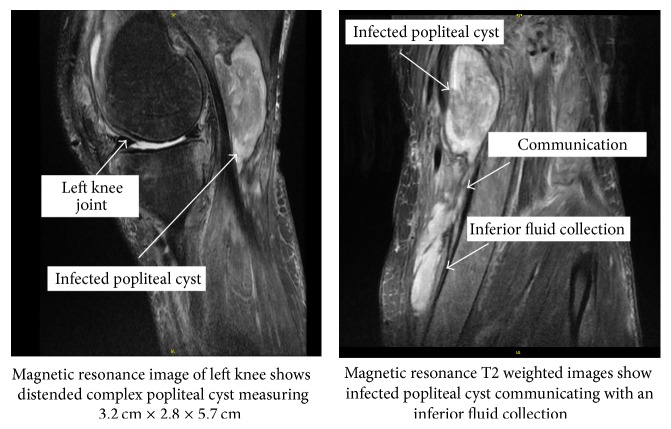
MRI images of left knee and infected fluid collections.

About 6 weeks after the arthroscopic procedure, patient developed fever with recurrence of pain and swelling in left knee while he was on vacation. He declined any surgical intervention at the time and was prescribed oral amoxicillin-clavulanate 875–125 mg twice daily. Upon his return, he was evaluated in the orthopedic surgery clinic and was noted to have a large, firm, indurated subcutaneous mass measuring about 5-6 inches in diameter in the posteromedial aspect of left knee. Magnetic resonance imaging (MRI) showed extensive soft tissue edema around the knee with multiple complex peripherally enhancing fluid collections (Figure 1).

About 30 ml of purulent material was aspirated with ultrasound guidance and anaerobic culture grew* Actinomyces meyeri*. He did not have any cough or productive sputum. Patient improved briefly with aspiration and 10-day-course of oral amoxicillin-clavulanate 875–125 mg twice daily.

Few weeks later, he was referred to the Infectious Diseases Clinic when his symptoms recurred and was noted to have erythema, warmth, and induration involving the posterolateral aspect of his left calf, without any fluctuance, purulent drainage, or sinus tract. Ultrasound of left leg showed complex popliteal cystic fluid collection with internal debris extending into posterior lateral calf. 20 ml of purulent material was aspirated with ultrasound guidance and its analysis showed 533,000 cells per cubic millimeter; RBC 114,000 cells per cubic millimeter; 98% segmented neutrophils; 1% lymphocytes; and 1% eosinophils. Aerobic culture of aspirate was negative; anaerobic culture grew* Actinomyces meyeri* that was susceptible to penicillin and clindamycin. White Blood Cell count was 15.6 × 10(9)/L and CRP was elevated at 158 mg/L. Chest radiography was negative for any pulmonary infectious process. He was treated with 6 weeks of intravenous penicillin G 24 million international units every 24 hour via continuous infusion. Patient improved clinically and was transitioned to oral penicillin VK 500 mg twice daily.

**Figure 2:**
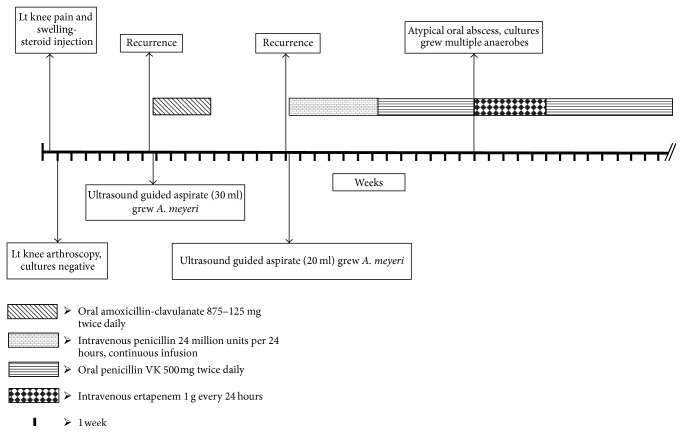
Timeline of clinical course and management.

While on oral penicillin regimen, patient developed severe throat pain. He was noted to have inflamed left posterior tonsillar fossa, atypical abscess with suspected fistulization, suggestive of actinomycosis. He underwent incision and drainage of left peritonsillar abscess and anaerobic culture grew multiple anaerobes but* Actinomyces meyeri* was not isolated. He received approximately 5 weeks of intravenous ertapenem 1 g every 24 hours, a broad spectrum antibiotic to cover multiple oral anaerobes until the left peritonsillar abscess resolved and was then transitioned to oral penicillin VK 500 mg twice daily (Figure 2).

Patient completed 12 months of oral penicillin VK treatment and he has not had any recurrence of symptoms 12 months after completion of treatment.

## 3. Discussion

Popliteal cyst infection is a rare infectious disease process that is usually an extension of an infected knee joint [1–8] and only a small number of cases have been reported in the medical literature [1, 4]. Clinical presentation may resemble deep venous thrombosis or cellulitis [5–7]. Ultrasonography usually reveals a cystic fluid filled collection in the posterior aspect of the knee. MRI is better in assessment of rupture of the cyst and associated pyomyositis or osteomyelitis.

To identify the pathogen and to determine the specific pathogen-targeted antimicrobial therapy, aspirate of cystic fluid should be sent for gram stain, bacterial cultures, Acid Fast Bacilli stain, mycobacterial culture, fungal stain, and fungal culture.* Staphylococcus aureus* appears to be the most common causative pathogen [1]. Only one case report of anaerobic popliteal cyst infection has been reported in the medical literature [6].

In our patient, anaerobic culture of the aspirate grew* Actinomyces meyeri* that was susceptible to penicillin.* Actinomyces* are part of the normal flora of the oral, gastrointestinal, and genital tract in human beings. They can become pathogenic when there is disruption in mucosal barrier, resulting in rare chronic infections (actinomycosis) involving oral/cervicofacial, intra-abdominal, and genitourinary tracts.

*Actinomyces israelii* is the most common member of genus* Actinomyces* to cause human infections, predominantly localized in cervicofacial diseases [9]. Other human pathogens include* A. naeslundii, A. viscosus, A. odontolyticus, A. gerencseriae, A. meyeri, A. europaeus, A. neuii, A. radingae, A. graevenitzii, A. turicensis, A. georgiae, A. funkei, A. lingnae, A. houstonesis, and A. cardiffensis* [9].* A. meyeri* is an uncommon cause of actinomycosis in humans. Only 34 cases of infections by* Actinomyces meyeri* have been reported in English language literature when search was done in December 2016 [10–12].

Unlike* A. israelii*,* A. meyeri* usually causes pulmonary infection [13–16] and has been noted to have propensity for systemic disseminated disease involving the heart [17], bones [18, 19], liver [20–22], spleen [23], brain [24, 25], and muscles [26]. Localized infections in the jaw [27], breast [28], disk space [29], skin [30], orbit [31], foot [32], or abdomen [11, 33] also occur if there is direct inoculation due to injury or surgery. A review of cases noted that more than one-third of patients with* A. meyeri* had evidence of gingival/dental infection and/or alcohol use [10]. About half of patients with pneumonia were noted to have evidence of systemic dissemination [10].

*Actinomyces meyeri* is thought to cause primary pulmonary infection with subsequent local empyema and distant haematogenous dissemination to brain, liver, or spleen. A significant number of patients have poor dental hygiene and history of alcoholism, suggesting that aspiration to the lungs is the primary inciting event in the pathogenesis [10].

Our patient did not have any evidence of dental abscess at the time of presentation with popliteal cyst infection. He had history of dental abscess approximately 4 months prior to the presentation. About 4 months after the initial presentation, he was noted to have peritonsillar abscess which required incision and drainage. The peritonsillar abscess fluid culture grew multiple anaerobic organisms but* Actinomyces meyeri* was not isolated. We hypothesize that the portal of entry of* A. meyeri* in our patient is likely oral mucosa with subsequent hematogenous dissemination to the left knee popliteal cyst without pulmonary involvement. He received intra-articular steroid injection during his initial visit which might have exacerbated the infectious process. Patient did not have any clinical or radiological evidence of pulmonary infection. The infectious process extended beyond the popliteal cyst likely either as a result of partial rupture of the cyst into the surrounding soft tissue in the posterolateral aspect of the left leg or due to tissue invasion by* Actinomyces*.* Actinomyces* do not respect any tissue boundaries and can easily invade tissues to spread infection beyond the popliteal bursa. The initial arthroscopy noted cloudy fluid but joint fluid cultures were negative.

Treatment requires a lengthy course of antibiotic therapy along with adequate drainage or surgery.* A. meyeri* is susceptible to penicillin and disseminated disease is usually treated with intravenous penicillin, 18–24 million units per day, for two to six weeks, followed by penicillin v potassium or amoxicillin. In patients that cannot take penicillin, doxycycline or clindamycin is a viable alternative. The duration of antibiotic therapy is variable and can range from six to twelve months, pending clinical response [10].

In conclusion, infections of popliteal cyst due to* A. meyeri* are very uncommon. Periodontal disease and alcoholism are risk factors for infection with* A. meyeri*.* A. meyeri* has a predilection for disseminated disease which could be secondary to more frequent pulmonary infection. A penicillin-based regime remains the treatment of choice and a relatively long course is needed. Surgical or percutaneous drainage of abscesses is recommended, as needed. The overall prognosis is fair, even in the presence of disseminated disease.

## Additional Points

*Learning Points*. They were as follows:

- Popliteal cyst infection is a very uncommon complication that can resemble cellulitis or deep venous thrombosis.
- *Actinomyces meyeri* is part of oral microflora and an uncommon cause of infection in humans.
- *Actinomyces israelii* usually causes localized cervicofacial disease where as* Actinomyces meyeri* is noted to cause pulmonary infection with propensity to cause systemic disseminated disease involving skin, long bones, muscle, liver, and brain.
- *Actinomyces meyeri* is sensitive to penicillin. Current recommendation based on expert opinion is IV penicillin for 2–6 weeks followed by prolonged 6–12 months course of oral penicillin VK. The total duration of antibiotics should be individualised based on clinical and radiological progress.
